# Development of a protein microarray-based diagnostic chip mimicking the skin prick test for allergy diagnosis

**DOI:** 10.1038/s41598-020-75226-y

**Published:** 2020-10-23

**Authors:** Marina Kalli, Andrew Blok, Long Jiang, Nichola Starr, Marcos J. C. Alcocer, Franco H. Falcone

**Affiliations:** 1 Molecular Therapeutics and Formulation Division, School of Pharmacy, University of Nottingham, Nottingham, UK; 2 Advanced Materials and Healthcare Technologies Division, School of Pharmacy, University of Nottingham, Nottingham, UK; 3 School of Biosciences, University of Nottingham, Nottingham, UK; 4 Institute for Parasitology, Justus-Liebig-University of Giessen, Biomedizinisches Forschungszentrum Am Seltersberg, Schubertstr. 81, 35392 Giessen, Germany

**Keywords:** Lab-on-a-chip, Materials for devices, Diagnosis, Fluorescence imaging

## Abstract

Protein microarrays have been successfully used for detection of allergen-specific IgE in patient sera. Here, we demonstrate proof-of-concept of a solid-phase technique coupling the high-throughput potential of protein microarrays with the biologically relevant readout provided by IgE reporter cells, creating a novel allergic sensitization detection system. Three proteins (κ-casein, timothy grass pollen extract, polyclonal anti-human IgE) were printed onto three different polymer-coated surfaces (aldehyde-, epoxy- and NHS ester-coated). ToF–SIMs analysis was performed to assess printed protein stability and retention during washing steps. NFAT-DsRed rat basophil leukemia cell attachment and retention during washing steps was assessed after treatment with various extracellular matrix proteins. NFAT-DsRed IgE reporter cells were sensitized with serum of an allergic donor, incubated on the printed slides, and cell activation determined using a microarray laser scanner. NFAT DsRed IgE reporter cell binding was significantly increased on all polymer surfaces after incubation with fibronectin and vitronectin, but not collagen or laminin. All surfaces supported printed protein stability during washing procedure, with epoxy- and NHS ester-coated surfaces showing best protein retention. Cell activation was significantly higher in NHS ester-coated slides after timothy grass pollen extract stimulation appearing a suitable substrate for further development of an automated allergy diagnosis system.

## Introduction

Allergy is defined as an immune-mediated hypersensitivity reaction mainly initiated by an IgE-dependent immunological response to otherwise innocuous antigens (allergens). The prevalence and severity of allergic diseases has dramatically increased, with 300 million people diagnosed with asthma, and a similar number of people (200–250 million) suffering from food allergy^1^.

Immunoglobulin E (IgE) antibody concentration is the lowest of the five immunoglobulin subtypes (IgA, IgG, IgM, IgD, IgE) found in human serum (50–300 ng/mL^−1^)^2^. However, its immunostimulatory potency is amplified by the high affinity receptor FcɛRI on mast cells and basophils, to which it binds. Crosslinking of the FcɛRI/IgE complex by a multivalent allergen triggers a signal transduction cascade, ultimately leading to an immediate hypersensitivity reaction, consisting of the release of preformed mediators which are present in cell granules such as histamine, serine proteases (tryptase and chymase), and the de novo synthesis and secretion of cytokines, chemokines and arachidonic acid metabolites (leukotrienes, prostaglandins) that attract and/or activate inflammatory cells^3^.

Allergies are typically diagnosed initially by the taking of a detailed medical history, followed by a physical examination. This is complemented by specific skin prick tests (SPT) and serum immunoassays, which are performed to detect and quantitatively measure allergen-specific IgE (sIgE) antibodies in human serum^4^.

Throughout the twentieth century, allergic diseases were studied using allergen extracts for diagnostic and therapeutic purposes^5^. However, standardization was difficult, due to inconsistencies in source materials and production processes, which led to considerable variation in results^6,7^.

Microarray technology has allowed the simultaneous screening of patient serum samples in a miniaturized scale against more than one hundred suspected protein allergens, used as recombinant proteins, for the first time^8–10^. This technology enabled detection of binding of sIgE to different individual allergen components—a process referred to as component resolved diagnosis (CRD)^11^. One important advantage of CRD to the previous allergen extract technology is the ability to distinguish between genuine sensitization and cross-reactivity, also allowing identification of the so-called ‘molecular spreading’ phenomenon^12^. However, a major limitation of the allergen-specific IgE reporting assays is that they do not necessarily document the crosslinking of the FcɛRI/IgE complex on the surface of basophils and mast cells by allergens, which is closely associated with allergy clinical symptoms^13^. Lin et al. proposed the coupling of protein arrays with peripheral blood basophilic cells, thus showing a biological readout could be a potential tool in diagnosis of allergic sensitization^14^. However, purifying basophils from peripheral blood of human donors is not a widely acceptable proposition for non-specialised labs, despite strongly improved and semi-automated protocols for their purification^15^. We and others have previously suggested replacing human basophils with humanised rat basophilic leukaemia (RBL) reporter cell lines^16–19^. RBL cells were first introduced in 1981 and have been used for many decades as a mast cell model to investigate the molecular basis of IgE-dependent signal transduction^20^. Earlier methods relied on the relatively insensitive measurement of beta-hexosaminidase release and were thus dependent on the use of a cell sensitivity enhancing agent such as deuterated water (D_2_O)^21^ or 5′-N- ethylcarboxamide (NECA)^22^. Nakamura R. et al*.* were the first to develop a reporter system named EXiLE, for IgE crosslinking (x)-induced luciferase expression, after stably transfecting humanised RBL SX-38 cells^23^ with a nuclear factor of activated T cells (NFAT)-responsive reporter construct^19^. Activation of the RS-ATL8 reporter cells relied on measuring firefly luciferase reporter gene expression using a chemiluminescent substrate. This system could be used in 96- or 384-well format^18^. It would be ideal to combine such a cellular, biologically relevant readout with the array format of CRD. However, RS-ATL8 cells and the ExiLE system are not compatible with an array format, as lysis of the cells is required prior to substrate measurement. Therefore, an alternative reporter system expressing a fluorescent reporter gene such as DsRed was developed and recently improved by our group^16,18^.

Here, we further this developed method and present proof-of-concept for a novel type I allergy diagnosis system that allows the simultaneous determination of sIgE combining purified allergen molecules printed on glass slides with fluorescent humanised RBL reporter cell lines.

## Results

### Comparative profiling of cell adhesion on ECM-treated polymer-coated surfaces

**Figure 1:**
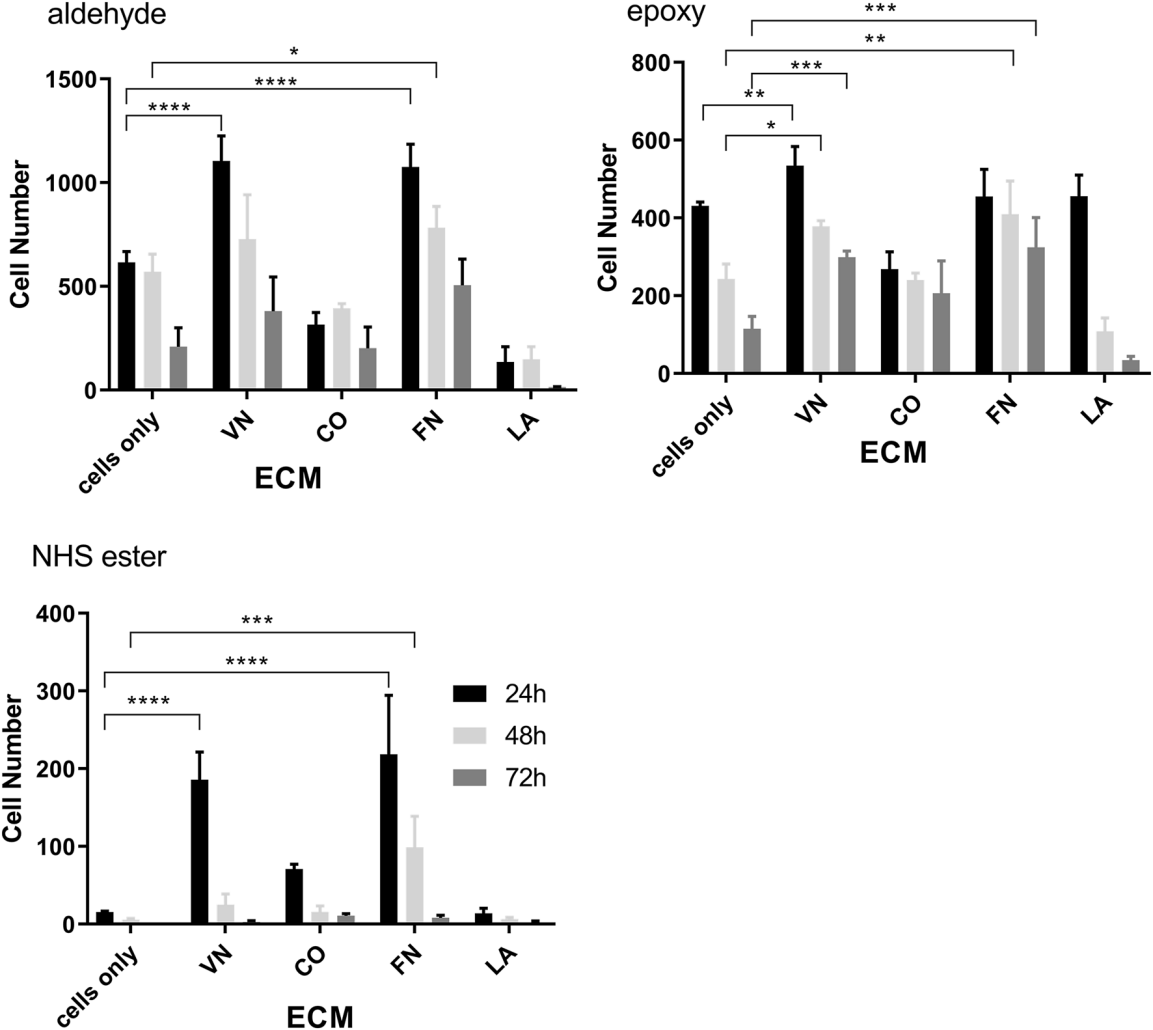
RBL 703–21/NFAT-DsRed cell attachment on aldehyde-, epoxy- and NHS ester-coated surfaces treated with vitronectin (VN), collagen (CO), fibronectin (FN), laminin (LA) or no treatment (cells only), after 24 (black bars), 48 (light grey bars) and 72 h (dark grey bars). Data are means ± SEM (n = 3). *p < 0.05; **p  < 0.01; ***p < 0.001, or ****p < 0.0001 (2-way ANOVA followed by Tukey’s multiple comparisons test).

In this study, three different surface chemistries (aldehyde-, NHS ester- and epoxy-coated) were compared for their ability to support and maintain adhesion of NFAT-DsRed IgE reporter cells using four different ECM proteins (vitronectin, fibronectin, laminin and collagen). The number of cells attached after different incubation periods (24, 48 and 72 h) are shown in Fig. 1.

Treatment of aldehyde-, epoxy- and NHS ester-coated surfaces with vitronectin and fibronectin caused significantly increased cell retention after 24 h. Cell attachment was also increased after 48 h, and 72 h for fibronectin-treated NHS ester-coated surfaces, and for both vitronectin- and fibronectin-treated epoxy-coated surfaces. In contrast to NHS ester-coated slides, aldehyde- and epoxy-coated slides promoted good cell attachment also in the absence of ECM proteins (‘cells only’). NHS ester-coated slides showed the lowest levels of cell attachment even in the presence of vitronectin and fibronectin. Overall, fibronectin and vitronectin supported significant cell attachment on all three surfaces, compared to laminin and collagen.

### ToF–SIMS ion distribution maps of printed proteins for assessment of printed protein stability

To confirm the presence of the printed test proteins before and after washing the surfaces with deionized water, surface analysis of the protein microarrays using ToF–SIMS was performed. The ability of the three surface chemistries to bind and retain spotted protein before and during the washing procedure, carried out to remove non-adherent protein, was assessed for the three test proteins, i.e. polyclonal goat anti-human IgE (α-IgE; positive control), Timothy grass pollen extract (TGP-X; allergen test sample) and κ-casein (κ-casein; negative allergen control). Characteristic secondary ion fragments cleaved off from amino acids for mass spectrometric analysis of proteins as described by Saleem and Galla were used as a reference when examining the generated ToF–SIMS spectra to detect the presence of these proteins^24^.

**Figure 2:**
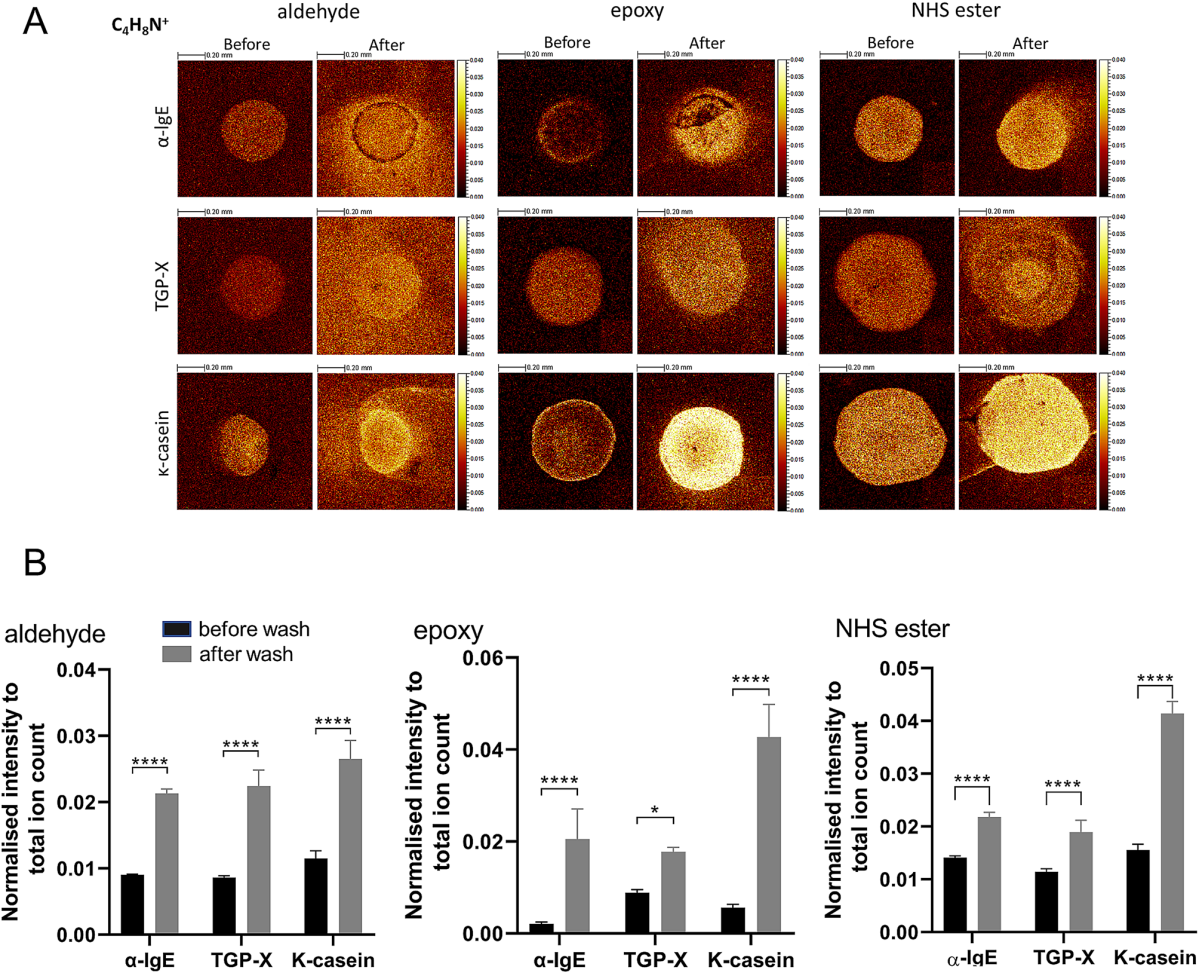
(**A**) ToF–SIMS ion distribution maps for the characteristic proline fragment (*m/z* 70 C_4_H_8_N^+^) on aldehyde-, epoxy- and NHS ester-coated surfaces. Each image was normalised to the corresponding total ion map. Brighter colour corresponds to higher normalised ion intensity. (**B**) Statistic Plots displaying normalised proline (C_4_H_8_N^+^) ion intensities of the three proteins (anti-human IgE, timothy grass pollen extract and κ-casein) printed on aldehyde-, epoxy- and NHS ester-coated surfaces. Data expressed are mean ± SD from quadruple determinations. Asterisks show results of Two-way ANOVA followed by Šídák’s multiple comparisons test; *p < 0.01, ****p < 0.0001.

ToF–SIMS ion distribution maps of characteristic amino acid fragments help revealing the presence of printed proteins. Each protein was printed using a 500 μm-sized ceramic pin on aldehyde-, epoxy- and NHS ester-coated surfaces. ToF–SIMS examines printed proteins on the three different surfaces before and after the washing. Normalised ion images of a characteristic proline fragment (m/z 70, C_4_H_8_N^+^) are displayed in Fig. 2A indicating the distribution of proteins.

Additional amino acid ions analysed of normalised ion images of characteristic glycine (m/z 30, CH_4_N^+^) and alanine (m/z 44 C_2_H_6_N^+^) fragments are presented in S1 Fig. (Supplementary information). In general, clear and defined protein dots were found for all printed proteins before the washing procedure, except for lgE on epoxy-coated slides, which showed a less homogeneous pattern. After washing, a more intense protein signal was noticed for all samples, which is also clearly confirmed in the corresponding statistical plots in Fig. 2B. Considering that buffer salts can potentially cause negative effects in ToF–SIMS analysis such as masking protein signals^25^, we determined salt ion distribution before and after washing as well. Na^+^ was chosen to illustrate salt distribution before and after the washing step. It is evident from S2 A and S2 B Fig. that Na^+^ intensity decreased after washing on all surfaces, hence, confirming the shielding hypothesis. Figure 2A also shows diffusion of proteins on substrates after washing procedure. This appeared greatest on aldehyde-coated slides. Figure 2B shows that after the washing step, all three substrates show a similar proline intensity for anti-human IgE (α-IgE) and timothy-grass pollen extract (TGP-X), while the epoxy surface shows a much higher proline intensity for κ-casein.

### Detection of activated RBL cells on polymer coated surfaces

**Figure 3:**
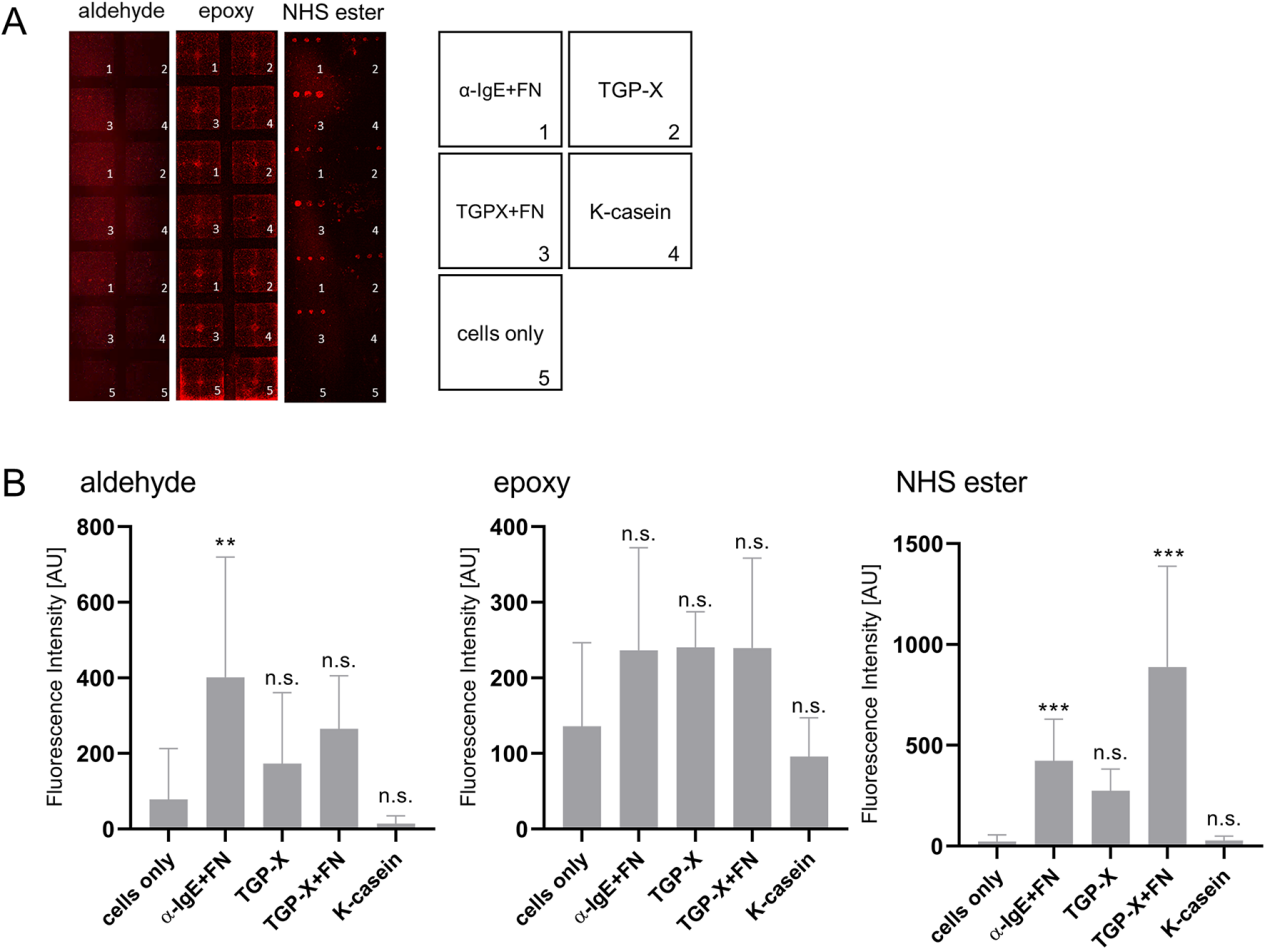
(**A**) Cell binding of NFAT-DsRed IgE reporter cells to immobilized proteins printed in triplicate [(**1**) anti-human IgE + 10% fibronectin (FN), (**2**) timothy grass pollen extract (TGP-X), (**3**) TGP-X + 10% fibronectin (FN), (**4**) κ-casein and (**5**) cells only] on aldehyde-, epoxy- and NHS ester-coated slides. RBL cells were sensitised with the serum of a timothy grass pollen allergic patient overnight and incubated with the slides (8.4 × 10^5^ cells/slide) for 24 h. Images of each protein spot were scanned using a GenePix 4000B laser scanner at 635 nm. (**B**) Fluorescence intensity values derived from each activated protein-spot on aldehyde-, epoxy- and NHS ester-coated slides after allergen-specific cell activation. Spot fluorescence intensity values were compared to negative control (cells only) and collected in triplicate. Data analysis was performed using one-way ANOVA, followed by Dunnett's test. P values < 0.05 were considered statistically significant (*p < 0.05; **p  < 0.01; ***p < 0.001, or ****p < 0.0001).

NFAT-DsRed IgE reporter cells were sensitized overnight with serum from a donor who is allergic to timothy grass, but not to milk. Next day, sensitised cells were added to the slides onto which timothy grass pollen extract (TGP-X; with or without fibronectin), as well as a positive (anti-IgE plus fibronectin) and a negative control (κ-casein) had been printed and incubated for a further 18 h. After this final incubation, cell activation on each surface was determined using a microarray laser scanner at 635 nm and a fluorescence microscope using RFP channel (Exc: 531 nm/Em: 593 nm). The raw fluorescent scanner images in Fig. 3A show that the surfaces supported cell activation to varying degrees. Images revealed distinct round-shaped dots especially on NHS ester- and to a lesser extent on aldehyde-coated surfaces, displaying successful fluorescent reporter gene activation on the printed spots. Epoxy-coated surfaces showed the least activation; furthermore, this material showed high background noise, as evidenced by the consistent high fluorescence intensity spots in the middle and on the edges of every well, which are not related to activated cells. GenePix software was used to quantify the fluorescence intensity generated from each individual protein-spot.

As shown in Fig. 3B, NHS ester-coated slides show a consistent activation pattern with timothy grass pollen + 10% fibronectin (TGP-X + FN) having the highest activation, followed by anti-human IgE + 10% fibronectin (α-IgE + FN), timothy grass pollen extract (TGP-X) and κ-casein showing levels similar to negative control (‘cells only’). Timothy grass pollen + 10% fibronectin fluorescence intensity in NHS ester-coated surfaces is 3 × higher compared to aldehyde- and epoxy-treated surfaces. On the aldehyde- and epoxy-coated slides, the fluorescence intensity observed was highly variable between spots. Epoxy-coated slides showed high fluorescence intensity levels in wells without any printed protein (cells only) and the lowest intensity values. Overall, this surface seemed to be more prone to artefacts (see activation on edges of incubation chamber in Fig. 3A).

**Figure 4:**
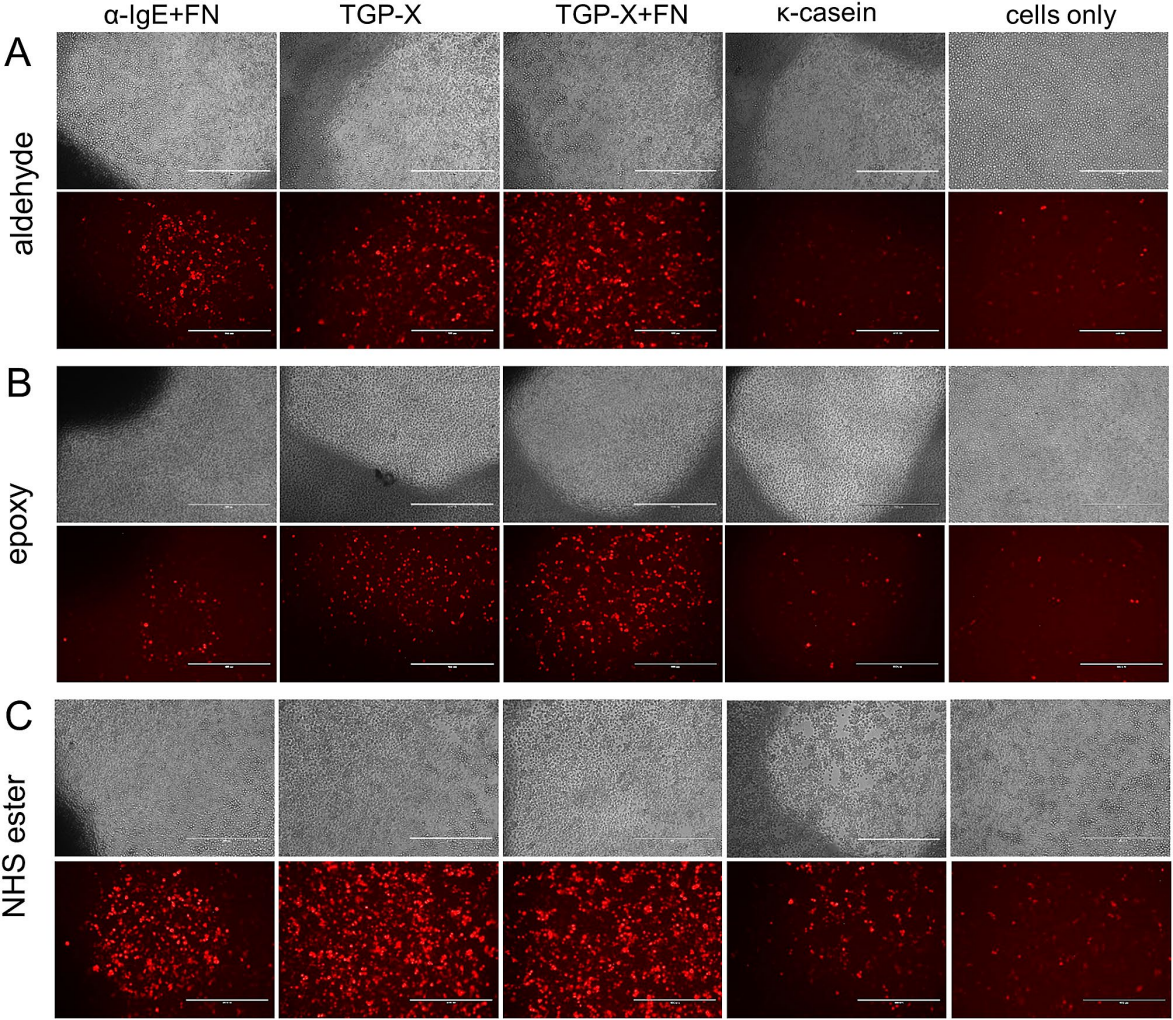
Cell activation images of NFAT-DsRed IgE reporter cells immobilized onto anti-human IgE, timothy grass pollen extract (TGP-X) with and without Fibronectin (FN) or κ-casein printed on aldehyde-, epoxy- and NHS ester-coated surfaces. Images of each protein spot were captured using fluorescence light microscopy (RFP channel) with a 10 × objective lens. Image scale bar 400 μm.

Allergen-specific activation of the NFAT-DsRed IgE reporter cells was confirmed by fluorescence microscopy using the RFP channel (531 nm excitation, 593 nm emission). As shown in Fig. 4, timothy grass pollen extract plus 10% fibronectin (TGP-X + FN) resulted in higher RBL cell activation compared to cells only (no protein) on all surfaces. Image comparison showed higher DsRed protein expression on NHS ester-coated surfaces compared to aldehyde- and epoxy-treated ones. However, weak cell activation was also observed in the ‘cells only’ slides (no protein), which most likely resulted from spontaneous and/or surface-induced activation.

**Figure 5:**
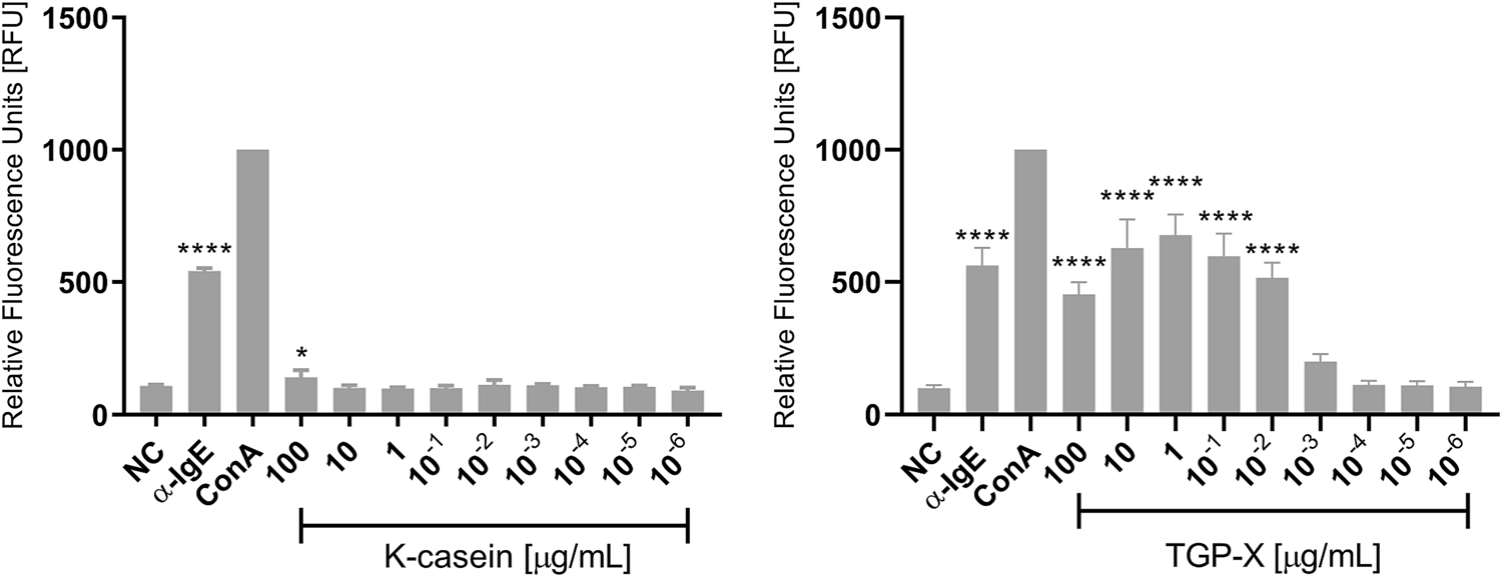
Fluorescence of NFAT-DsRed in conventional 96-well format. Anti-human IgE, ConA (both 1 μg/mL; used for data normalization) and timothy grass pollen extract (TGP-X) or κ-casein (both 1 pg/mL to 100 μg/mL) were added to reporter cells sensitized overnight with grass pollen allergic donor serum allergic. After a further 18 h, to allow for production and maturation of fluorescent reporter protein DsRed in activated cells, the plates were measured in a Tecan Infinite M200 plate reader using 530 nm excitation, and 590 nm emission.

Our final set of experiment were to assess activation using the same materials (patient serum and proteins) used for the array format using the NFAT DsRed IgE reporter in the conventional 96-well soluble phase format, to rule out any artefacts caused by the different coated array surfaces. NFAT DsRed cells were sensitised overnight with allergic patient serum as before, and the allergens (TGP-X and κ-casein) added in a wide concentration range from 1 pg/mL to 100 μg/mL the next day. After a further 18 h of incubation, fluorescence was measured using a fluorescent plate reader. As shown in Fig. 5, results obtained from the 96-well format were very similar to those obtained with the NHS ester-coated arrays.

While TGP-X gave a positive test result in the concentration range between 10 ng/mL and 100 μg/mL, indicating the presence of timothy grass pollen-specific IgE in the patient’s serum, κ-casein gave negative results in all tested concentrations, except for a small but statistically significant (*p* < 0.5) positive signal at the highest concentration 100 μg/mL. Both positive controls anti-IgE and ConA (the latter was used for normalization of data, allowing direct comparisons between different plates) gave strong positive signals.

## Discussion

Advances in biochip technology has led to the development of valuable tools for quantitative measurement of allergen-specific IgE levels in allergic individuals^26^. More specifically, protein microarrays provide a miniaturized allergy test where hundreds to thousands of purified proteins are applied onto glass slides to diagnose allergic sensitization using only small amounts of patient sera. However, allergy diagnosis is not fully mirrored by measuring allergen-specific IgE in vitro, as merely IgE binding to the high affinity IgE receptor FcεRI does not always correlate with clinical symptoms. This is illustrated by the fact that skin prick tests and RAST test frequently remain positive even in individuals who have lost (‘outgrown’) clinical sensitivity to the offending foods. Another complication in the context of food allergies is the existence of so-called cross-reactive carbohydrate determinants (CCDs), which bind to IgE without having the ability to activated basophils or mast cells by crosslinking receptor-bound IgE (recently reviewed by Homann et al*.*^27^).

To overcome these limitations, Lin et al. (2007) proposed coupling of protein arrays with effector basophilic cells as a feasible method to diagnose allergy using a cellular readout^14^. Toward this end, basophilic cell activation results in over-expression of surface markers that could be linked with an allergic response. However, a number of limitations such as the low abundance of human peripheral blood basophils (< 1% of total white blood cells), the need for basophil purification and the large volumes of patient sera to isolate the required amount of cells, rendered the practical application of this system difficult^14^.

This suggested that basophil cell lines could be used as surrogate of patient basophils. The introduction of humanised rat basophilic leukaemia cell lines (RBL) stably transfected with the human FcεRIα chain, consisted a promising alternative as human IgE could bind with high efficiency to induce cell activation upon crosslinking with a matching allergen (see recent review^17^). First attempts using CD63 (a cell surface glycoprotein, member of the transmembrane 4 superfamily) as cell surface activation marker were unsatisfactory, due to high pre-activation levels of CD63 on RBL cells^14^. As stated earlier, the use of the RS-ATL8 reporter cell line that relies on NFAT-dependent expression of a firefly luciferase reporter gene, although highly sensitive, is not suitable to be tested in a microarray format. Measurement of luciferase activity would require cell lysis, thus resulting in fluorescent signal disconnection from the allergen spots^17^. Experiments performed with cell penetrating luciferase substrate formulations were attempted, but not further pursued. As a viable alternative, we developed a reporter system expressing intracellular fluorescence (RBL 703/21 NFAT-DsRed) showing high stability in solid-phase assays^16^.

In order to establish proof-of-principle that our fluorescent reporter cell line could be used in printed array format, we first needed to investigate several parameters regarding printed protein stability before and after washing the surfaces, cell binding to solid surfaces, and the ability of different polymer coatings to support cell activation. The ideal material would support high protein binding and retention during washes, promote cell attachment and importantly, not induce toxic effects. The two first properties are fulfilled by the widely used FAST slides^28^ which were used in the original work by Lin and her co-authors^14^. These slides have a very high binding capacity resulting in near quantitative protein binding and retention. However, we found the proprietary nitrocellulose matrix of FAST to have strong toxic effects during the necessary overnight incubation with RBL cells and were therefore not suitable for our purpose. Thus, we needed to explore further surface chemistries that would provide good protein binding but also promote long term binding of cells, without toxicity.

Cell attachment onto the polymer surfaces is facilitated by the absorption of extracellular matrix proteins onto the polymer slides. In addition to attachment, ECM proteins facilitate diverse cell functions such as cell growth, adhesion and migration, and mediate the indirect interaction of cells with the polymer^29^. Most importantly, use of FN together with the test allergen improved the signal-to-noise ratio from 11.7 to 37.9 (positive control: 18), with high statistical support. Thus, the use of FN appears necessary to achieve reliable detection.

In our experiments, the aldehyde- and epoxy-activated glass slides supported RBL cell binding during the first 48 h, regardless of the presence of ECM proteins (Fig. 1A,B). However, RBL cell binding was significantly increased after treating all surfaces with fibronectin (FN) and vitronectin (VN), whereas no significant binding was observed after collagen (CO) and laminin (LA) treatment. These results were expected, as nearly forty years ago, a number of studies demonstrated that fibronectin and vitronectin, are two key serum glycoproteins that facilitate attachment and spreading of culture cells^30–32^ via a tripeptide sequence, arginine–glycine –aspartic acid (RGD) which allows a specific interaction with α5β1 and α_v_β3 integrin cell surface receptors in fibronectin and in vitronectin, respectively^33^.

Similarly, Ra et al.^34^ showed that mast cells attached more prominently to FN and VN rather than CO and LA. Wang et al. also reported that RBL 703/21 cells attached strongly to FN coated surfaces but also, although less prominently, to CO and LA^16^. Other authors^35,36^ have also reported similar findings on RBL-2H3 cell binding to collagen type I and laminin.

In order to confirm the presence of the printed proteins before and after washing the surfaces with de-ionised water, we performed ToF–SIMS analysis that enables label-free detection of individual molecules^24^. Recently, ToF–SIMS has drawn interest, especially as a means of examining bio/organic surfaces, owing to its surface sensitivity and chemical specificity^37^. More specifically, ToF–SIMS is a surface-sensitive chemical analysis technique, where a primary ion beam (Bi^3+^ in this study) is used to bombard sample surface inducing a collision cascade that produces secondary ions^38^. The emitted secondary ions are then collected in a time-of-flight analyser and separated based on their mass versus charge (m/z) ratio for identification of molecular species on the surface. For proteins, small mass ions are detected which usually correspond to fragments from the 20 proteinogenic amino acids. To interpret low mass fragmentation patterns, researchers have identified characteristic ions that correspond to individual amino acids^39^. However, protein identification can be challenging, since all proteins contain the same 20 amino acids, unique peaks from adsorbed proteins cannot be easily differentiated^40^. Alternatively, multivariate methods such as principal component analysis (PCA), provide detailed interpretation of spectral data by reporting differences between each spectrum (scores) which can then be related to the different fragmentation pattern of the spectra (loadings)^41–43^.

Nonetheless, for the purposes of this study, we used only the characteristic peaks of individual amino acid fragments generated by ToF–SIMS to identify printed proteins. The characteristic peak of the essential amino acid proline (C_4_H_8_N^+^) was chosen as a marker to illustrate the presence of the proteins. As shown in Fig. 2A,B, all surfaces were suitable to use with protein microarrays. Washing procedure is expected to move loosely bound protein and buffer salts. The increased intensity of proline ion (C_4_H_8_N^+^) distribution (Fig. 2A) is attributed to the removal of buffer salt, which is confirmed by the distribution of Na^+^, a chosen marker of buffer salts. It is clear from S2 Fig. B (Supplementary information), that Na^+^ ion intensity levels were higher before the washing step and greatly decreased after washing in all three surfaces tested, leading to increased signal detection of amino acid-related ion fragments. Diffusion patterns of proteins on different substrates (Fig. 3A), however, indicate that aldehyde is less able to retain the bound protein during washes.

Next, after testing RBL cell activation on three different polymer-coated surfaces, we observed that the NHS ester-treated surfaces had larger printed surface diameters, which we assumed would have an effect on cell binding and ultimately on cell activation. This was expected, as the (NHS ester) CodeLink activated slides are prepared using a hydrophilic polymer containing N-hydroxysuccinimide ester groups. According to the slide manufacturers, due to the hydrophilic nature of the coating, the spot size would be slightly larger than that achieved with coatings that are more hydrophobic^44^. Kimzey and co-authors, after printing GFP fluorescence protein on various chemistry surfaces from different manufacturers, reported that N-hydroxysuccinimide (NHS)-ester coated slides were able to bind significantly higher amount of protein compared to epoxide and aldehyde coated surfaces^45^. Yang et al*.* reported similar findings, after examining the relationship between the cell number and the surface chemistries analysed by ToF–SIMS^46^. They concluded that ToF–SIMS ions corresponding to the tertiary butyl moiety (C_4_H_9_^+^) and phenyl group (C_6_H_5_^+^) showed lower cell adhesion, whereas NHS ester-treated surfaces with the characteristic ion C_3_H_8_N^+^ resulted in high cell adhesion^46^.

In this study, we report for the first time fluorescent RBL reporter cell activation on allergen-printed arrays. Our data provide proof-of-principle data that the proposed system can be used to detect the presence of allergen-specific IgE in the tested sera. The serum used here was from a clinically well-characterised donor with high levels of specific IgE (equivalent to RAST class IV or higher) to several grass and tree pollens, but without any allergy to milk proteins.

While the technology presented here is still in need of an extensive validation using well-characterised clinical samples, there are a few notable advantages. In comparison to e.g. UniCAP or related techniques, where sIgE levels are measured to individual allergens, the array format allows printing of allergens in high densities. This would allow for example the creation of allergen arrays customised to specific regions of the world, in which different sets of allergens (pollens, food, etc.) are clinically relevant. Allergens printed on arrays are very stable and can be stored at room temperature for long periods of time. The key difference however is in the entity measured: while UniCAP and similar technologies measure the amount of sIgE bound to the tested allergens immobilised on a solid phase, the array technology presented here does not measure sIgE directly, but measures the cellular activation response induced by allergen-dependent cross-linking of sIgE on the cell surface. As such, it is less prone to artifacts caused by the aforementioned cross-reactive carbohydrate determinants (CCDs), which can result in false positive test results. At the same time, the signal transduction pathway provides a powerful signal amplification system which is not present in traditional sIgE measurements without cells. Finally, all humanized IgE reporter cell systems, whether performed in multiwell- or array-format, include washing steps after overnight sensitization with the patients’ serum. These washes result in the removal of large amounts of IgG, as the cells are not transgenic for human IgG receptors, while IgE will bind with high affinity to the human IgE receptor. Any competition for the same epitope(s) between IgG and IgE is therefore avoided, which is likely to result in increased sensitivity. For example, the EXiLE technology, developed by Nakamura et al.^19^ can detect a measurable response to as little as 1 fg/mL of egg white protein in serum of egg white-allergic patients.

Our results are in agreement with the serological tests and the conventional, soluble phase assay with the NFAT DsRed reporter, showing significantly increased fluorescence intensity only to anti-human IgE (positive control) and timothy grass pollen extract (test allergen) but not to κ-casein (negative control allergen). Our future efforts will be geared towards the assessment of multiple allergens using well-characterised patient sera to confirm the robustness of our newly developed system in allergy diagnosis, and ultimately determine key parameters such as specificity, sensitivity as well as negative and positive predictive value.

## Conclusion

Collectively, our results prove the feasibility of developing an allergy diagnosis system using humanised reporter cell lines in combination with allergens printed in array format. By testing a limited number of different polymer coatings representing the main chemistries available, we were able to identify NHS ester-coated slide as a very suitable material for this technology, allowing good cell adhesion, promoting cell activation with low background and no toxicity.

## Methods

### Cell culture

All experiments were performed with rat basophilic leukaemia cells (RBL-703/21-derived NFATp-DsRed-Express2) generated at The University of Nottingham^16^. These are available from the authors for non-commercial purposes on the basis of a material transfer agreement (MTA). Cells were grown in Minimum Essential Medium Eagle’s (EMEM) cell culture medium supplemented with 10% v/v heat-inactivated foetal bovine serum (Gibco, UK), 100 U/mL penicillin, 100 μg/mL streptomycin and 2 mM L-glutamine (Merck, UK) in a 37 °C/5% CO_2_ humidified cell incubator. To maintain stable expression of FcεRIα_H_, cells were cultured with 1 mg/mL G418 sulphate (Thermo Fisher Scientific, UK) and 20 μg/mL Blasticidin S HCL (InvivoGen, USA) for selection of NFATp-DsRed-Express2 reporter gene expression.

### Protein printing

The following proteins or protein extracts were diluted in Dulbecco’s phosphate-buffered saline (DPBS) to a final concentration of 1 mg/mL, polyclonal goat anti-human IgE (Merck, UK), *Phleum pratense* (timothy grass) pollen extract (kindly donated by Dr Gabriele Schramm, Research Centre Borstel, Germany) and κ-casein (Merck, UK), chosen as a common, well known cow’s milk allergen^47^ to which the tested serum donor is not sensitized, containing 10% FN and timothy grass pollen extract w/o FN, were printed in triplicate onto the functionalised slides using a XYZ3200 dispensing workstation (Biodot, US) and 0.5 mm Xtend microarray ceramic pins (LabNEXT Inc, US). The printing conditions were O_2_ < 2000 ppm, 25 °C, and 34% humidity. The arrays were dried at room temperature (RT) for 24 h.

### Time-of-flight secondary-ion mass spectrometry (ToF–SIMS)

Analysis of the printed proteins before and after washing with DI water was conducted using a ToF–SIMS IV instrument (IONTOF GmbH, Münster, Germany) with a Bi^3+^ cluster primary ion source operated at 25 kV. The primary ion dose density was maintained at < 1 × 10^12^ ions/cm^2^ to ensure static conditions. Positive polarity spectra were acquired in high current bunched mode over 1 × 1 mm areas at a resolution of 228 pixels per mm, using the macroraster stage function. Retrospective data analysis allowed ions indicative of amino acids (e.g., CH_4_N^+^, C_2_H_6_N^+^, C_4_H_8_N^+^) to be identified. Positive spectra were mass calibrated using CH_3_^+^ (m/z 15), C_2_H_5_^+^ (m/z 29), C_3_H_7_^+^ (m/z 43), and C_4_H_9_^+^ (m/z 57) peaks. Data analysis was carried out using SurfaceLab 7 software (IONTOF GmbH, Münster, Germany).

### Cell adhesion assay

Glass slides functionalised with the following three chemistries: Aldehyde- (Nanocs, UK), Epoxy- (Molecular Devices/Genetix, UK) and CodeLink activated Amine-binding/NHS ester-coated (Surmodics, US). The exact chemical nature of the polymer coatings is proprietary. The alkylsilane molecules can be terminated with different reactive groups, e.g. aldehyde-, epoxy- or amine-reactive maleimide groups. On the Nanocs aldehyde slide, the aldehyde density is 50 ~ 100 μmol CHO/cm^2^. Proteins are covalently attached via a Schiff’s base reaction with their N-terminal NH_2_ group and NH_2_-groups in the side chains. CodeLink slides (now called TRIDIA) are coated with a hydrophilic polymer containing N-hydroxysuccinimide (NHS)-ester reactive silane groups, which bind proteins via amino groups. We were unable to find any additional information regarding the Genetix epoxy-coated silane slides. Epoxy-groups are highly reactive and result in covalent linkage of proteins via their amino-, thiol- or hydroxy-groups in a ring opening reaction.

Slides were treated with five different ECM proteins diluted in Ca^2+^/Mg^2+^-free DPBS (Merck, UK) to a final concentration of 10 μg/mL. Non-functionalised glass slides were used as the negative control. Adhesion assays were carried out in 16-well ProPlate Multiwell Chambers (Grace Bio Labs, US) in sterile Petri dishes (100 × 15 mm diameter) containing 200 μL of each ECM. The functionalised glass slides were coated with vitronectin (VN) (Advanced BioMatrix, US), laminin (LN) from Engelbreth-Holm-Swarm murine (Merck, UK) and fibronectin (FN) from human plasma (Merck, UK) in a 37 °C/5% CO_2_ humidified cell incubator and collagen type I solution from rat tail (CO) (Roche Diagnostics, UK) at 4 °C for 48 h. Prior to the assay, the incubated glass slides were washed three times with sterile DPBS.

Cultured cells were collected using trypsin/EDTA (Merck, UK) for 15 min in a 37 °C/5% CO_2_ humidified cell incubator. The cells were resuspended in fresh medium to a final concentration of 6 × 104 cells/200 μL. They were then added in each chamber-well and kept at 37 °C for a maximum of three days. Every 24 h, 100 μL (1 μg/mL) Hoechst 33342 solution (ThermoFisher Scientific) dissolved in DPBS was added for 20 min at 37 °C. After incubation, cells were washed once with sterile DPBS and 200 μL EMEM was added to each well. Cell adhesion was assessed using an Evos *fl* fluorescence microscope. Images were taken at 10 × magnification in bright field and blue fluorescence using the DAPI light cube (Exc: 357 nm, Em: 447 nm). Cell number and image analysis was performed using Fiji ImageJ V 1.6.

### Cell activation assay

RBL cells were seeded at a concentration of 6 × 10^4^ cells/200 μL in each well of 6-well tissue-culture treated plates (Corning, UK). After seeding, cells were sensitized with 20 μL/mL (1:50 diluted in cell culture medium) serum of a grass pollen allergic donor for 16 h. Ethical Approval was granted by the University of Nottingham School of Pharmacy Research Ethics Committee (Ref. 047-2018). All methods were carried out in accordance with relevant guidelines and regulations. Blood donors gave their written informed consent.

The next day, printed surfaces were sterilised using ultraviolet light in a Class II biosafety cabinet at 30,000 μJ/cm^2^ for 30 min. Following this, surfaces were washed three times with 0.05% v/v Tween 20 (Merck, UK) in DPBS. Next, they were blocked with 100 μL bovine serum albumin (Merck, UK) diluted in DPBS to a final concentration of 1 mg/mL. After 1 h incubation at 37 °C, they were washed 3 × with 0.05% v/v Tween 20 and 1 × with sterile DPBS before coating with 100 μL FN for 3 h at 37 °C. After incubation, the slides were washed 3 × with DPBS. Sensitized cells were collected using sterile cell scrapers (Corning, UK) and 200 μL cell suspension was added in each well of the 16-well multiwell chambers for 24 h in a 37 °C/5% CO_2_ humidified cell incubator. The next day, cell activation was assessed using an Evos *fl* Digital Inverted fluorescence microscope at 10 × magnification, using the RFP channel (531 nm excitation, 593 nm emission). The developed slides were then scanned with a GenePix 4000B scanner (Axon Instruments Inc., Union City, CA, USA) using a 635 nm laser, followed by image analysis with GenePix Pro 6.1 software (Axon Instruments Inc.). The fluorescence intensity of each cell-protein spot was calculated by subtraction of the local background from the mean intensity of the spot. For cell activation experiments in 96-well format, NFAT DsRed cells were used as described by Wan et al.^48^.

### Statistical analysis

Statistical analysis of cell activation was performed using one-way ANOVA, followed by Dunnett's post hoc test. Cell binding was analysed using two-way ANOVA, followed by Tukey’s post-hoc test compared with negative controls cells only without treatment, for each individual time point. *P* values < 0.05 were considered statistically significant (*p < 0.05; **p < 0.01; ***p < 0.001, or ****p < 0.0001).

## Supplementary information

Supplementary Information
