# Supplementary material for: Development of a protein microarray-based diagnostic chip mimicking the skin prick test for allergy diagnosis
Source: Sci Rep. 2020 Oct 23;10:18208. doi: 10.1038/s41598-020-75226-y (PMC7584649; doi:10.1038/s41598-020-75226-y)
Supplement: Supplementary file 1 — Supplementary Information [file 41598_2020_75226_MOESM1_ESM.pdf]

Supporting Information

Development of a Protein Microarray-Based Diagnostic Chip Mimicking the Skin Prick Test for Allergy Diagnosis. Marina Kalli, Andrew Blok, Long Jiang, Nichola Starr, Marcos J.C. Alcocer and Franco H. Falcone

ToF-SIMS Ion Distribution Maps for Glycine and Alanine

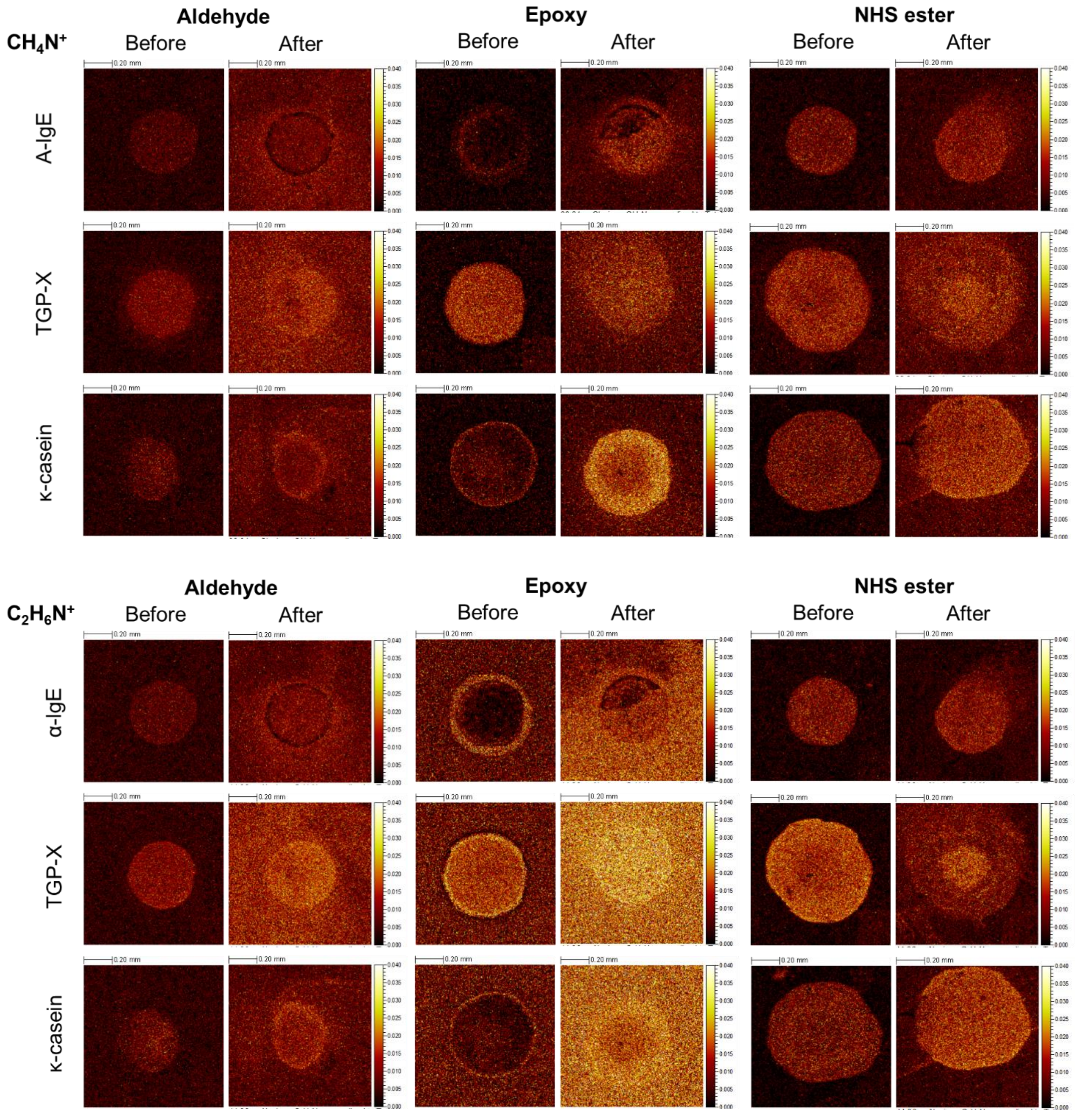

S1 Fig. ToF-SIMS ion distribution maps for the characteristic glycine (*m/z* 30, CH_4N^+) and alanine (*m/z* 44 $C_2H_6N^+$) fragments on aldehyde-, epoxy- and NHS ester-coated surfaces. Each image was normalised to the corresponding total ion map. Brighter colour corresponds to higher normalised ion intensity.

S2 Fig. Na⁺ Ion Intensity Distribution

A

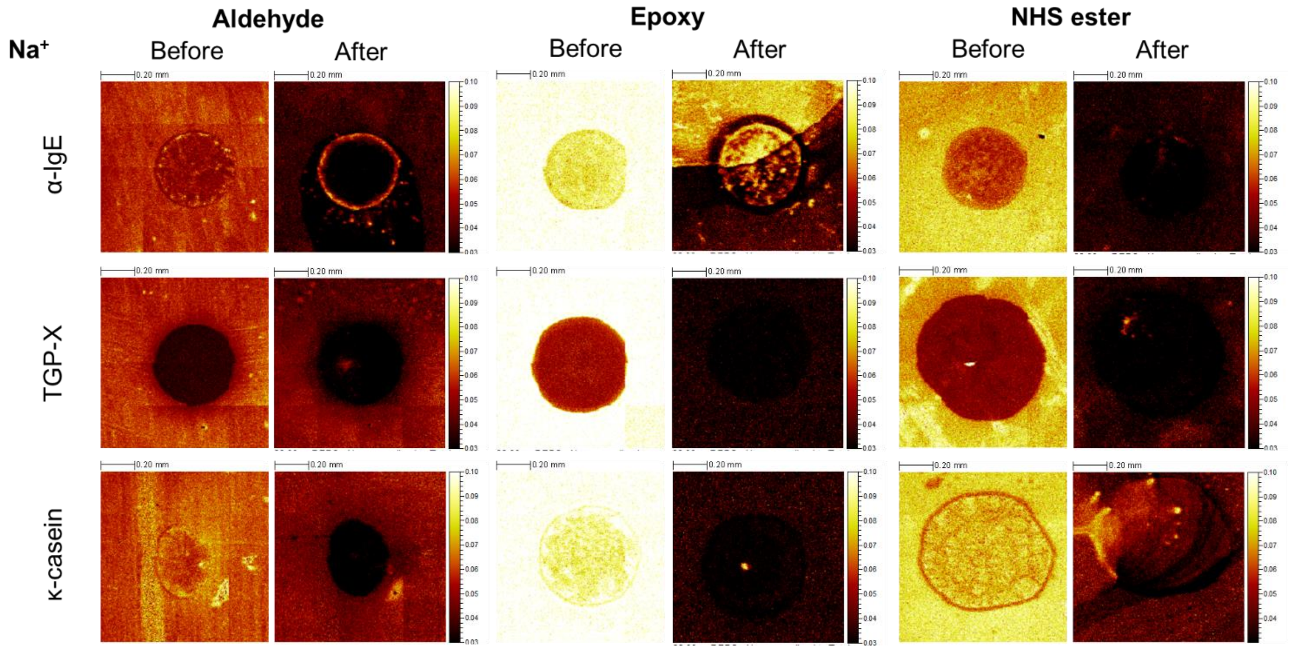

B

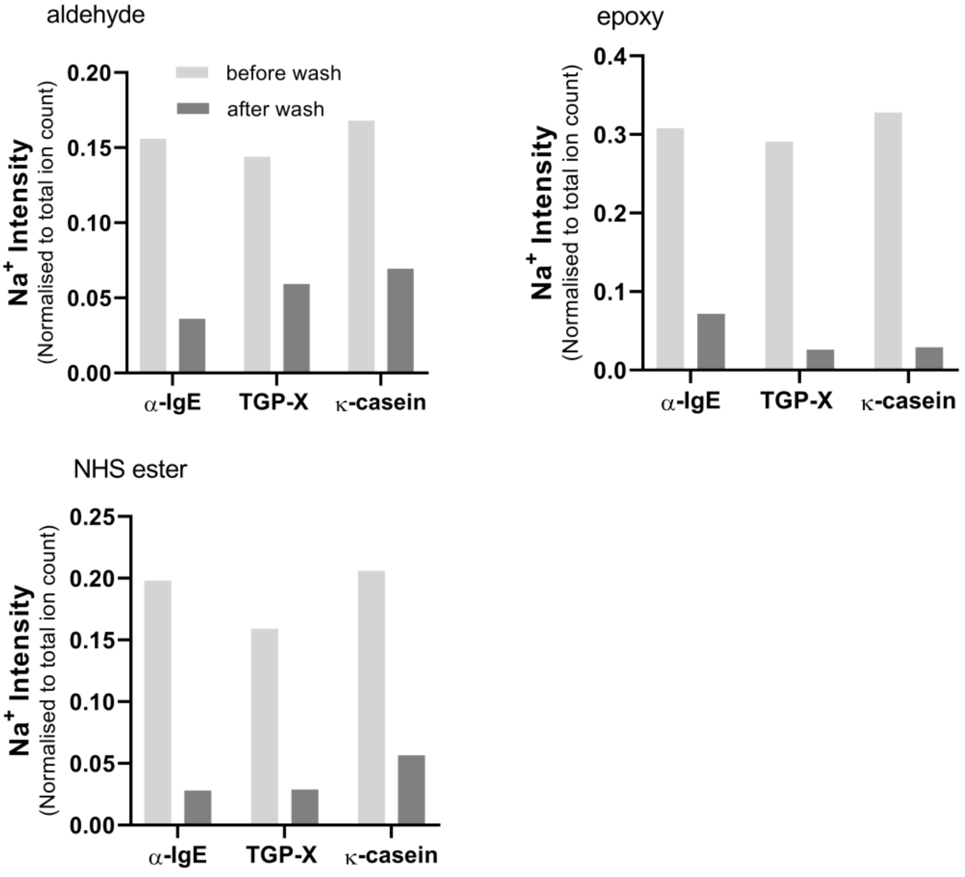

S2 Fig. ToF-SIMS ion distribution maps for the characteristic sodium fragment (*m/z* 22.99 Na^+) on aldehyde-, epoxy- and NHS ester-coated surfaces. Each image was normalised to the corresponding total ion count. Brighter colour corresponds to higher normalised ion intensity. **B.** Bar charts displaying normalised sodium (Na^+) ion intensities of the three proteins (α-IgE, timothy grass pollen extract and κ-casein) printed on aldehyde-, epoxy- and NHS ester-coated surfaces. Data presented derive from single ion intensity measurements.
